# The Protective Effect of Snail Secretion Filtrate in an Experimental Model of Excisional Wounds in Mice

**DOI:** 10.3390/vetsci8080167

**Published:** 2021-08-20

**Authors:** Enrico Gugliandolo, Francesco Macrì, Roberta Fusco, Rosalba Siracusa, Ramona D’Amico, Marika Cordaro, Alessio Filippo Peritore, Daniela Impellizzeri, Tiziana Genovese, Salvatore Cuzzocrea, Rosanna Di Paola, Patrizia Licata, Rosalia Crupi

**Affiliations:** 1 Department of Veterinary Science, University of Messina, 98166 Messina, Italy; egugliandolo@unime.it (E.G.); fmacri@unime.it (F.M.); plicata@unime.it (P.L.); rcrupi@unime.it (R.C.); 2 Department of Chemical, Biological, Pharmaceutical and Environmental Science, University of Messina, 98166 Messina, Italy; rfusco@unime.it (R.F.); rsiracusa@unime.it (R.S.); rdamico@unime.it (R.D.); aperitore@unime.it (A.F.P.); dimpellizzeri@unime.it (D.I.); tgenovese@unime.it (T.G.); 3 Department of Biomedical and Dental Sciences and Morphofunctional Imaging, University of Messina, 98166 Messina, Italy; cordarom@unime.it; 4 Department of Pharmacological and Physiological Science, Saint Louis University School of Medicine, Saint Louis, MO 63104, USA

**Keywords:** snail secretion filtrate, *Helix aspersa* muller, wound management

## Abstract

Wound healing is a physiological process comprising several coordinated phases, such as inflammation, proliferation, and remodeling. For centuries, *Helix aspersa* Muller mucus has been known to have biological properties that are useful for treating skin disorders. In this study, we used a full-thickness excisional wound model in mice to test the hypothesis that Snail Secretion Filtrate (SSF) can improve the wound healing process. The mucus from *Helix aspersa* Muller was obtained mechanically by manually stimulating snails with a sterile cotton swab tip, and then the mucus was subjected to a series of filtrations to obtain SSF. After wounding, the mice were treated topically with SSF for 14 days. Our macroscopic results show that the SSF treatment significantly improved the speed and percentage of wound area closure. Furthermore, SSF improved several markers of proper wound healing, such as collagen deposition (Masson, COL3A1, matrix metalloproteinases (MMPs)) and the tissue remodeling process (α-sma, vascular-endothelial growth factor (VEGF)). SSF was also able to counteract the inflammatory process in injured wound tissue (myeloperoxidase (MPO) IL-1β, IL-6, TNF-α). In conclusion, our results show that SSF is able to enhance the speed and efficiency of wound healing and positively regulate several aspects of the wound healing process, such as the proliferative and remodeling phases.

## 1. Introduction

Wound healing is an important physiological process that maintains the integrity of skin after trauma resulting from accidents, external factors, medical procedures, or other causes. In both humans and animals, wound healing is a complicated process comprising several phases, including inflammation, proliferation, and remodeling [1]. Normally, the result of these well-coordinated sequential phases is the complete healing of the wound with the restoration of a normal skin structure. However, in the presence of different pathological factors, such as an underlying disease state or infection, the wound can become chronic. A chronic wound can be defined as a wound that is stalled at a certain point of the standard physiological process of wound healing [2].

For centuries, *Helix aspersa* Muller mucus has been known to have biological properties that are useful for treating skin disorders, and recently, its use has received considerable interest and has been proposed for the formulation of para-pharmaceutical products for wound management and as a constituent of cosmetic products [1]. The snail produces mucus with several functions necessary for survival. The mucus, which covers the external surface of the snail, is produced by particular secretory epidermal glands and has adhesive, emollient, antimicrobial [3], and reparative properties [4,5]. Despite the increased interest in the use of this substance, there is currently a lack of scientific data on its biological effect, the molecular mechanism of the effect, and its chemical characteristics, such as the chemical composition, as snail mucus can vary among different species and breeding conditions [5]. The mucus from *Helix aspersa* Muller is characterized by the presence of mucopolysaccharide, hyaluronic acid, polyphenols, and several other bioactive molecules and minerals [6]. This particular composition and the presence of mucopolysaccharide have been observed to improve the adhesion of the mucus to the skin and, thus, act as a barrier, and the presence of polyphenols provides the mucus with the ability to counteract oxidative damage. Furthermore, *Helix aspersa* Muller mucus is able to stimulate the synthesis of endogenous hyaluronate and, thus, increase the water-binding capacity and viscoelasticity of the skin [6].

Recently, we focused on the biological properties of crude Snail Secretion Filtrate (SSF). Mucus was obtained manually by stimulating snails with a sterile cotton swab tip and then subjected to a series of filtrations to obtain SSF. In our previous study, we performed a full chemical characterization of SSF and demonstrated its protective effect in a mouse model of an ethanol-induced gastric ulcer [7]. We found that, in addition to the high content of mucopolysaccharide, SSF is characterized by high levels of glycolic acid and collagen, followed by allantoin and elastin, and by the presence of other biological molecules such as vitamin A, groups B and E, and several minerals, including copper, nickel, and chromium. Our previous study showed that the protective effect of SSF in an ethanol-induced gastric ulcer model in mice is mediated by an increase in the preservation of mucosal mucopolysaccharides and collagen. Oxidative stress and the induced inflammatory response were also reduced [7].

In this study, we investigated the effect of SSF in a full-thickness excisional wound model in mice. The effect of SSF on the wound healing process was evaluated with a focus on different molecular mechanisms, such as the collagen pattern (Masson stain, COL3A1, MMPs), tissue remodeling (VEGF, α-sma, TGF-β), and the inflammatory process (MPO, mast cells, IL-1β, TNF-α).

## 2. Materials and Methods

### 2.1. Animals

CD1 mice (male, 20–30 g; ENVIGO, Indianapolis, IN, USA) were housed in a controlled environment in standard mouse cages (Tecniplast, Italy, Italy) (room temperature 22 °C and 12 h light/dark cycles) with standard rodent chow and water provided ad libitum. The animals were adapted to these conditions for 1 week. Messina University Review Board for the care of animals approved the research, and the approved project identification code is 294/2021-PR. All animal experiments were in accordance with the new regulations in Italy (D.Lgs 2014/26) and EU regulations (EU Directive 2010/63). All experiments were conducted in accordance with ARRIVE guidelines. 

### 2.2. Snail Secretion Filtrate (SSF) Collection and Sterilization

*Helix aspersa* Muller mucus was kindly provided by Snail S.R.L.S (Messina, Italy). Briefly, the breeding was cruelty-free. In particular, the mucus was obtained mechanically by manually stimulating snails with a sterile cotton swab tip. In the first step, the mucus was filtered with a coarse filter to stabilize the pH, after which the mucus was passed through a filtration train consisting of 3 different filters (10 microns, 1 micron, 0.22 micron; Pall) and then stored at 4 °C. The use of the 0.22-micron filter was necessary to eliminate impurities and endotoxins in order to make the product injectable. The full chemical characterization was previously performed [7].

### 2.3. Full-Thickness Excisional Wound Model

Mice were anesthetized with isoflurane (3% in air) prior to shaving the hair on their back areas, and then betadine scrub and 70% ethanol were applied alternately 3 times to prepare the dorsum for wounding. Two full-thickness excisional wounds (5 mm) were generated with a sterile biopsy punch (KAI Corporation, Tokyo, Japan) on the dorsum to create two circular full-thickness skin wounds on either side of the median line on the dorsum. Mice were randomly divided into the following groups:

SHAM *n* = 10: mice were subjected to all procedures described above except that the full-thickness excisional wounds were not applied.

CONTROL *n* = 10: Mice were subjected to full-thickness excisional wounds as described above. A topical application of 400 μL of sterile dH_2_O was administered with a micropipette once a day for 14 days.

SSF *n* = 10: Mice were subjected to full-thickness excisional wounds as described above. Wounds were treated once a day for 14 days with a topical application of 400 μL of SSF with a micropipette.

Each wound site was digitally photographed at the indicated time intervals, and the wound area was quantified using ImageJ software and was expressed as the percentage of the original wound size over time. Fourteen days after wounding, the mice were euthanized by cervical dislocation under anesthesia, and wound samples were harvested as follows: Using the residual wound as the center, a round skin sample (diameter, 10 mm) containing all the layers of skin was harvested [8]. In all mice, the left wound site was fixed in 4% formaldehyde for histological analysis, and the right wound site was immediately stored at −80 °C for further analysis.

### 2.4. Histopathological and Immunohistochemical Analyses

Wound specimens were fixed in 4% formaldehyde and then embedded in paraffin Sections were stained with H&E for histological analysis, the field was chosen to include all wound bed into the optical field magnification 10×. The histological score on a point scale from 0 to 4 was determined as previously described. Briefly, the scores were 0: absence of epithelial proliferation in >70% of the tissue; 1: poor epidermal organization in >60% of the tissue; 2: incomplete epidermal organization in >40% of the tissue; 3: moderate epithelial proliferation in >60% of the tissue; 4: complete epidermal remodeling in >80% of the tissue. Paraffin-embedded skin tissues with a thickness of 5 μm were stained with Masson’s trichrome according to the manufacturer’s protocol (Bio-Optica, Italy). The relative density of blue-stained collagen after Masson’s trichrome staining was quantified in the wound area using ImageJ, the field was chosen to include all wound bed into the optical field magnification 10×. Immunohistochemical analyses were performed as previously described. Briefly, the sections were incubated overnight with primary antibodies anti-α-sma (Santa Cruz Biotechnology, sc-130617, Heidelberg, Germany), anti-VEGF (1:250, Santa Cruz Biotechnology sc-57496, Heidelberg, Germany), and anti-MPO (1:250 Santa Cruz Biotechnology, sc-390109, Heidelberg, Germany) and processed as previously reported. Five stained sections from each mouse were scored in a blinded fashion and observed using a Leica DM6 microscope (Leica Microsystems SpA, Milan, Italy). The histogram profile was related to the positive pixel intensity value obtained, into wound bed with optical field magnification 10× or 40×.

### 2.5. ELISA

Expression of Collagen Type II Alpha (COL3A1) protein was quantified through ELISA assay (Cloud-Clone Corp., Katy, TX, USA) according to the manufacturer’s protocol.

### 2.6. Toluidine Blue Staining

Skin sections were deparaffinized in xylene and dehydrated by a graded series of ethanol, 5 min in each solution. The sections were then placed in water for 5 min, immersed in toluidine blue for 4 min, and then blotted carefully. Sections were placed in absolute alcohol for 1 min, cleared in xylene, and fixed on glass slides using Eukitt (Bio-Optica, Milan, Italy) as previously reported [9,10]. The number of metachromatically stained mast cells was determined by counting 5 high-power fields (40×) randomly chosen within each wound section using a DM6 microscope (Leica, Milan, Italy). Mast cells were identified using light microscopy by their metachromatic cytoplasmic granules. Elongated or oval metachromatic cells with a diameter of 10–30 μm, filled with granules sometimes overlying the oval nucleus, which were located centrally or eccentrically, were counted as mast cells. Portions mast cell cytoplasm, or clusters of metachromatic granules in the tissue were consistently discarded. The density of mast cells was expressed as the number of mast cells per unit area of wound bed tissue [11].

### 2.7. Western Blot

Western blot analyses were performed as previously described [12,13,14]. Briefly, specific primary antibodies anti-MMP1 (1:1000 Santa Cruz Biotechnology, sc-137044, Heidelberg, Germany), anti-MMP-2 (1:1000 Santa Cruz Biotechnology, sc-13595, Heidelberg, Germany), anti-MMP-9 (1:1000 Santa Cruz Biotechnology, sc-393859, Heidelberg, Germany), anti-IL-1β (1:1000 Santa Cruz Biotechnology, sc-52012, Heidelberg, Germany), anti-TGF-β (1:1000 Abcam), and anti-TNF-α (1:1000 Abcam) in 1× PBS, 5% (*w*/*v*) non-fat dried milk, and 0.1% TWEEN 20 were incubated with membranes at 4 °C overnight. Membranes were then incubated with peroxidase-conjugated bovine anti-mouse IgG secondary antibody or peroxidase-conjugated goat anti-rabbit IgG (1:2000; Jackson ImmunoResearch Laboratories, West Grove, PA, USA) for 1 h at room temperature. The levels of β-actin (1:2000; Santa Cruz Biotechnology) served as an internal control for protein loading [15]. The relative expression of the protein bands was detected with an enhanced chemiluminescence (ECL) system (Bio-Rad, Hercules, CA, USA) and visualized with the Chemi Doc XRS (Bio-Rad, Hercules, CA, USA). The bands were analyzed by using Image Lab 3.0 software (Bio-Rad, Hercules, CA, USA) and standardized to the relevant housekeeping protein level. A commercially available protein molecular weight marker (10–250 kDa) (Bio-Rad, Hercules, CA, USA) was used to define molecular weight positions and reference concentrations for each molecular weight.

### 2.8. Statistical Analysis

Each experiment included three independent replicates, and each experiment used *n* = 10. The data resulting from all experiments are expressed as means ± SEM. Statistical differences between groups were compared using unpaired t-test or one-way ANOVA, followed by Tukey’s post hoc test for multiple comparisons, using GraphPad Prism version 8 (GraphPad Software Inc., La Jolla, CA, USA). A *p*-Value of less than 0.05 was considered statistically significant.

## 3. Results

### 3.1. Snail Secretion Filtrate Accelerated the Rate of Wound Healing

**Figure 1:**
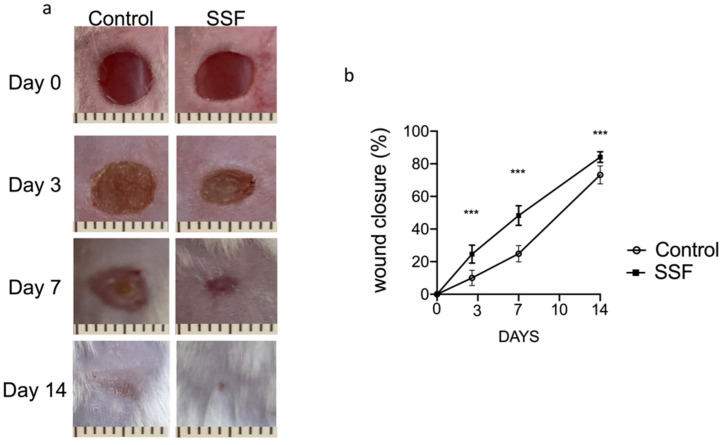
Effect of snail secretion filtrate (SSF) on the rate of wound healing in a mouse model of full-thickness excisional wounds. (**a**) Representative picture of wounds at the different time point (scale bar 1 mm). (**b**) The graph shows the results expressed as the percentage of original Wound size over time. Data are presented as means ± SEM images of 10 mice for each group. *** *p* < 0.001 versus control.

As shown by the picture in Figure 1, on day 0, we created full-thickness excisional wounds with a biopsy punch (5 mm). SSF was topically administered (400 μL) to the wound site for 14 days to test its potential therapeutic effects on the wound healing process. As shown by the pictures of wounds and the graph of wound closure as a percentage (Figure 1a,b), we observed that, compared to the control, daily SSF treatment significantly improved the wound healing process from days 3 to 14 in terms of speed and percentage of area closure.

### 3.2. Effect of SSF on Skin Wound Healing and Collagen Pattern

**Figure 2:**
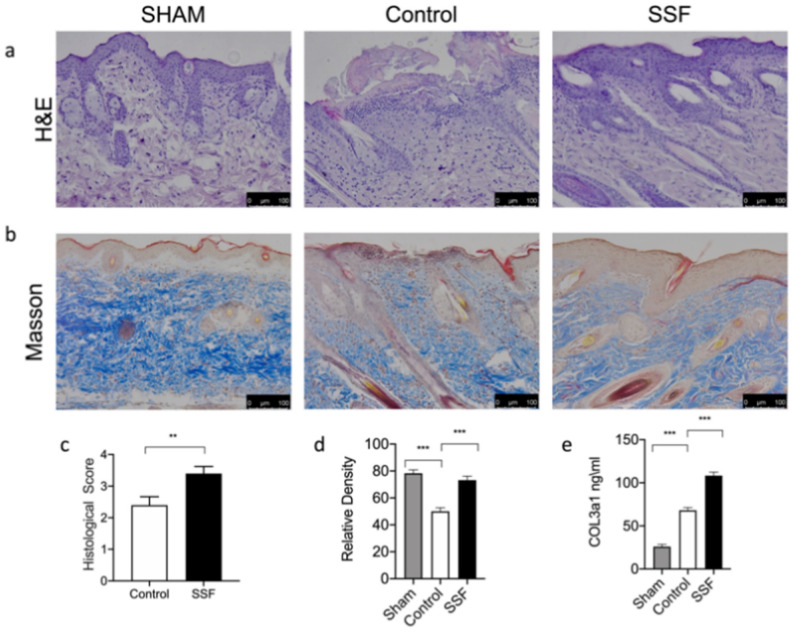
Effect of SSF on skin wound healing and collagen pattern in a mouse model of full-thickness excisional wound. (**a**,**c**) H&E staining of wound granulation tissue 14 days after wounding and relative histological score graph. (**b**,**d**) Evaluation of collagen deposition by Masson staining on day 14 after wounding and a relative density graph as a qualification of the stain intensity of blue collagen. (**e**) COL3a1 quantification by ELISA assay in wound tissue. Data are presented as means ± SEM images of 10 mice for each group. ** *p* < 0.01 *** *p* < 0.001.

As shown in Figure 2, histological examinations of wound tissue sections on day 14 after wound induction showed that the wound bed was still not completely covered by the epidermis in the vehicle control group, while in the group treated topically with SSF, the wound site showed an improvement in wound closure, granulation, and re-epithelialization, all of which contributed to a nearly complete restoration of the epidermis in this group. The histological score in Figure 2c, quantifies the re-epithelialization and, as shown by the graph, a significant increase was observed in the SSF-treated group 14 days after wound induction. To assess the effect of SSF treatment on the collagen content in wounds, Masson’s trichome staining was performed. As shown in Figure 2b, compared to the control, SSF treatment significantly improved the collagen content in the wound bed, restoring the collagen content to a level comparable to that in sham mice (Figure 2d). Then, we quantified the presence of COL3a1 in the different experimental groups. As shown by the graph in Figure 2e, the SSF treatment significantly increased the levels of COL3a1 14 days after wounding.

**Figure 3:**
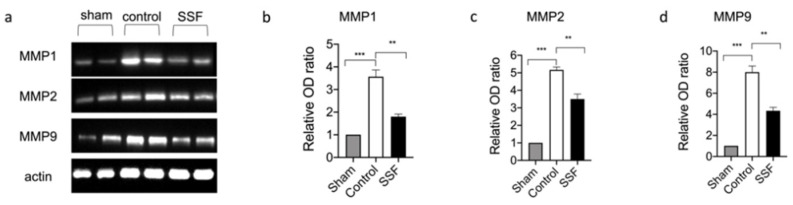
(**a**) Western blot analysis of wound tissue for matrix metalloproteinases MMP-1, MMP-2, and MMP-9, and relative densitometric analysis respectively. (**b**–**d**). Data are presented as means ± SEM images of 10 mice for each group. ** *p* < 0.01; *** *p* < 0.001.

Collagen deposition and maturation are regulated by several factors, such as the family of metalloproteinase (MMP). Through Western blot analysis, we evaluated the expression levels of the most important metalloproteinases involved in wound healing, namely, MMP-1, -2, and -9. As shown in Figure 3, the Western blot analysis of wound tissue for MMP-1, -2, and -9, demonstrates that, compared to the control, SSF was able to prevent the wound-induced overexpression of these factors.

### 3.3. Effect of SSF on α-sma and VEGF Expression on Wounds

**Figure 4:**
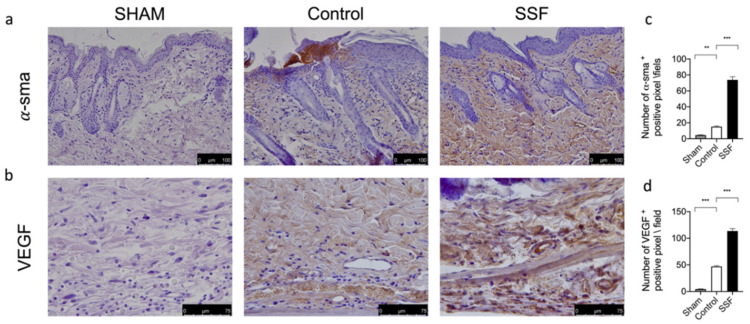
Effect of SSF on α-sma and vascular-endothelial growth factor (VEGF) expression. Immunohistochemical analysis of (**a**,**c**) α-sma and, (**b**,**d**) VEGF on wound bed tissue. Data are presented as means ± SEM images of 10 mice for each group. ** *p* < 0.01; *** *p* < 0.001.

Through a immunohistochemical analysis, we evaluated the expression of α-sma and VEGF, which are markers of a proper wound healing process, as VEGF is a key regulator of angiogenesis and α-sma a marker of cell differentiation. As shown in Figure 4, 14 days after wounding, the group treated daily with SSF showed a significantly increased expression of α-sma (Figure 4a,c) and VEGF (Figure 4b,d), indicating a proper wound healing and skin structure organization. 

### 3.4. Effect of SSF on Immune Cell Infiltration and Inflammatory Response 

**Figure 5:**
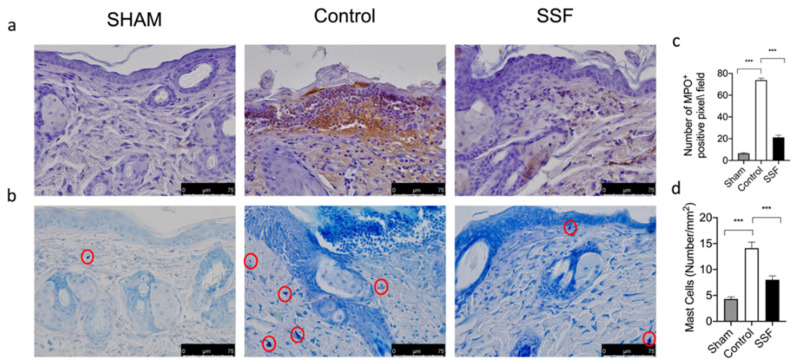
Upper panels (**a**,**c**) shows the immunohistochemical analysis of MPO in sham animals and in the wound bed 14 days after wounding. The group treated with SSF showed a significant decrease in MPO expression. Lower panels (**b**,**d**) shows the presence of mast cells in the wound bed, indicated by blue toluidine staining, from the sham group, control wound group, and wound + SSF treatment group, respectively. Data are presented as means ± SEM images of 10 mice for each group. *** *p* < 0.001.

Immune cell recruitment is a key event in the wound healing process. Thus, we evaluated the presence of inflammatory cells through the immunohistochemical analysis of MPO. As shown in Figure 5a,c, we found that the control group compared to the sham non-injured mice showed a significant increase in the presence of MPO^+^ cells, while treatment with SSF daily for 14 days significantly reduced the expressions of MPO in the wound bed. We also evaluated through blue toluidine staining the presence of mast cells, as another immune cell type involved in skin homeostasis. As shown by the pictures and relative graph in Figure 5b,d, the control group compared to the sham non-injured mice showed a significant increase in the presence of mast cells, while treatment with SSF daily for 14 days after wounding significantly decreased the presence of mast cells in the wound bed.

**Figure 6:**
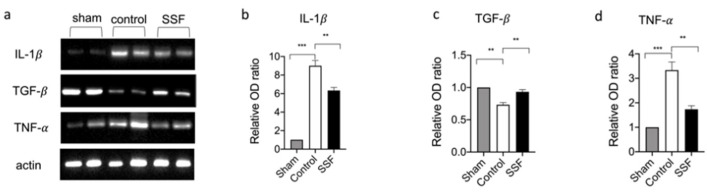
(**a**) Western blot analysis on wound tissue for. (**b**) IL-1β. (**c**) TGF-β, and (**d**) TNF-α, and relative densitometric analysis. Data are presented as means ± SEM images of 10 mice for each group. ** *p* < 0.01; *** *p* < 0.001.

Neutrophils and mast cells are immune cells involved in the inflammatory response. In particular, mast cells are sources of a wide variety of biologically active secreted products, including several cytokines and growth factors. We examined the cytokine levels in wound beds and found that 14 days after wounding, the control groups showed significantly high levels of expressions for the pro-inflammatory cytokines IL-1β and TNF-α (Figure 6a,b,d). Treatment with SSF topically for 14 days decreased the expression levels of IL-1β and TNF-α compared to the control group. As shown in Figure 6a,c, SSF treatment was also able to restore the level of the growth factor TGF-β in the wound bed. 

## 4. Discussion

Skin injuries are a very common condition that can occur as a consequence of several factors. Wound healing is a complex physiological process and consists of four integrated and overlapping physiological events: hemostasis, inflammation, proliferation, and tissue remodeling or resolution [16,17].

There is continuous interest in new strategies and compounds to enhance the wound healing treatment. In this regard, the benefit of snail mucus has been known for centuries in traditional medicine, but there is currently a lack of scientific knowledge on the chemical characterization and the molecular mechanism for the biological activity for this substance. Snail mucus-based compounds have especially received growing interest in cosmetology, particularly for their beneficial properties such as emollient, moisturizing, lubricating, and protective effects [18]. *Helix aspersa* Muller secretes a unique substance with important biological properties, and its protective effect on the skin has been attributed to its mechanical properties, mucopolysaccharide contents, and the specific composition of bioactive molecules [6]. In a previous study, we tested the protective effect of Snail Secretion Filtrate (SSF) in a mouse model of an ethanol-induced gastric ulcer. We also performed a chemical characterization of this substance [7]. We found that, in addition to its high content of mucopolysaccharide, SSF is characterized by high levels of glycolic acid and collagen, followed by allantoin and elastin, and by the presence of other biological molecules, such as vitamin A, groups B and E, and several minerals, including copper, nickel, and chromium. All of these compounds are known to have important biological properties, but the use of mucus has been demonstrated to have a greater effect than the isolated compounds [1].

In this study, we used a full-thickness excisional wound model in mice to test the hypothesis that SSF can improve the wound healing process. After wounding, the mice were treated with SSF for 14 days. Our macroscopic results showed that, compared to the untreated control mice, the SSF treatment significantly improved the speed and percentage of wound area closure. From a histological point of view, if compared to the control, we found that 14 days after wound induction in mice treated with SSF, the structure of the wound bed was well organized with a greater re-epithelialization. Collagen is a key factor in the wound healing process and a natural structural protein that is involved in all phases of the wound healing cascade [19]. In particular, collagen is a major compound in the extracellular matrix, and the regulated production, deposition, and maturation of new collagen is a key event in the wound healing process [20]. To highlight the presence of collagen, we performed Masson trichrome staining on the wound bed. Our results showed a significant increase in collagen deposition in the SSF-treated group compared to the control group. COL3A1, known as collagen α-1 type III, is present in extensible connective tissues such as the skin, lungs, and vascular system and plays a critical role in the wound healing and the cicatrization process [21]. Through ELISA assays, we quantified the presence of COL3A1 in the wound skin tissue. Our results showed significantly higher levels of COL3A1 in the SSF-treated group compared to the control group, and this result agrees with all results mentioned above, indicating that SSF has a significant pro-wound healing effect, as well as an important effect on the production and deposition/maturation of new collagen. These events are regulated by a family of proteins called Metalloproteinase (MMPs) [22], which play key roles in all stages of the wound healing process by modifying the wound matrix [23]. In particular, MMP-1, MMP-2, and MMP-9 play key roles in all stages of the wound healing process. The overexpression of MMPs impairs the remodeling and re-epithelization phases in wound-damaged models [24]. Our results showed that the SSF treatment was able to regulate the MMP expression by regulating the expression of MMP-1, MMP-2, and MMP-9. All of the above-described events, contributed to the formation of healthy granular tissue, a key event in the wound healing process. In particular, vascularization and wound contraction are a result of proper granular tissue formation and lead to better outcomes of the wound healing process [25].

A proper granular tissue formation is characterized by the presence of myofibroblasts, which can contract the granulation tissue and pull the edges of the wound closer to each other [26]. Thus, we evaluated the expression of VEGF in granular tissue as a regulating factor of angiogenesis in wound healing [27]. The upregulation of α-sma, which is a marker of myofibroblasts, promotes the differentiation of fibroblasts to myofibroblasts [27]. Our results showed that treatment with SSF significantly increased the expression of VEGF and α-sma and, thus, led to a more “efficient” formation of granular tissue. The high levels of α-sma could partly explain the high percentage of wound closure observed.

Although this event seems to be different across the different species previous studies have demonstrated that, although rodent wounds contract much more than other species, much of this contraction occurred only after epithelial closure and, thus, the simple excisional rodent wound model produced a well-defined and readily identifiable wound bed over which the process of re-epithelialization was clearly measurable [28].

The inflammatory process after injury is an essential physiological response. In the first phase after injury, the immune system is activated, initiating a local inflammatory response that includes the recruitment of inflammatory cells. However, a prolonged and unbalanced inflammatory response impairs wound healing [29]. Although the involvement of these immune cells is essential in the early stage of the wound healing process, excessive or prolonged activation of these cells can impair wound healing [29]. Our results showed that, in the control group, 14 days after wound induction, there was a significant presence of mast cells, as revealed by blue toluidine staining, and leukocytes, which were stained as MPO-positive cells. In particular, mast cells were identified using light microscopy by their metachromatic cytoplasmic granules stained with toluidine blue, although this methodology had some limitations [30]. For example, due to a lower number of mast cell profiles staining with toluidine-yielded compared to others’ staining techniques [31]. Treatment with SSF significantly reduced the presence of these immune cells infiltration in wound beds compared to the control group, but it did not completely inhibit the recruitment of immune cells, which were still higher than those in the sham group. In particular, mast cells are sources of a wide variety of biologically active secreted products, including diverse cytokines and growth factors [32]. Thus, we evaluated the expression of IL-1β, TNF-α, and TGF-β in wound beds 14 days after wounding. In particular, prolonged high levels of pro-inflammatory cytokines for several days or weeks are associated with impaired wound healing, in which the transition from the inflammatory to the proliferative phase of wound healing is inhibited [33]. Other mediators, such as growth factors, are essential for driving the wound healing process. Among such growth factors, TGF-β plays a key role in the wound healing process by regulating the inflammatory response, keratinocyte proliferation and migration, angiogenesis, collagen synthesis, and ECM remodeling [34]. Low levels of TGF-β in wounds have been associated with impaired wound healing [35]. Our results showed that, in agreement with the decreased presence of inflammatory cells, the SSF treatment significantly reduced the expression levels of the pro-inflammatory cytokines IL-1β and TNF-α. Furthermore, treatment with SSF increased the levels of the growth factor TGF-β, indicating the enhancement of a proper wound healing process.

## 5. Conclusions

In conclusion, our results showed that SSF was able to enhance the speed of the wound healing process. SSF positively regulated several aspects of the wound healing process, such as the inflammatory and proliferative phases. Due to the particular chemical composition of SSF, synergism among the activity of several molecules in this substance could not be excluded. We consider these compounds “in toto”, and it is not yet possible to confirm which molecule is responsible for the biological activity. Taken together, our results suggested that SSF could be a useful tool in wound management in both human and veterinary medicine. 

## Data Availability

All data obtained from this study are included in the manuscript.
